# First Person Perspective of Seated Participants Over a Walking Virtual Body Leads to Illusory Agency Over the Walking

**DOI:** 10.1038/srep28879

**Published:** 2016-07-01

**Authors:** Elena Kokkinara, Konstantina Kilteni, Kristopher J. Blom, Mel Slater

**Affiliations:** 1 Event Lab, Departament de Psicologia Clínica i Psicobiologia Faculty of Psychology, University of Barcelona, Spain; 2 Institució Catalana de Recerca i Estudis Avançats – ICREA, Spain; 3 Department of Computer Science, University College London, UK

## Abstract

Agency, the attribution of authorship to an action of our body, requires the intention to carry out the action, and subsequently a match between its predicted and actual sensory consequences. However, illusory agency can be generated through priming of the action together with perception of bodily action, even when there has been no actual corresponding action. Here we show that participants can have the illusion of agency over the walking of a virtual body even though in reality they are seated and only allowed head movements. The experiment (n = 28) had two factors: Perspective (1PP or 3PP) and Head Sway (Sway or NoSway). Participants in 1PP saw a life-sized virtual body spatially coincident with their own from a first person perspective, or the virtual body from third person perspective (3PP). In the Sway condition the viewpoint included a walking animation, but not in NoSway. The results show strong illusions of body ownership, agency and walking, in the 1PP compared to the 3PP condition, and an enhanced level of arousal while the walking was up a virtual hill. Sway reduced the level of agency. We conclude with a discussion of the results in the light of current theories of agency.

Normally humans are able to trivially distinguish their own actions from those of other people; we know we are the cause of our own volitional actions and take responsibility for the effects. This sensation of agency allows us to distinguish self-generated actions from those of others, contributing to bodily self-consciousness^1^,^2^. But how do we know that we ourselves produced a given action? In the study reported in this paper we show that it is possible to alter the perception of our actions, and create the illusory perception of carrying out an action (walking) while in fact seated.

Most theories of agency suggest that the brain predicts the sensory consequences of an action through an efference copy that takes as input the motor commands, and then compares predictions with the perceived outcomes. Hence, matches between the predicted sensory feedback (i.e. on the basis of the motor signals) and the sensory reafferences provide a sense of control over those actions – see the review by David, *et al*.^3^ and the earlier work by Holst and Mittelstaedt^4^, Sperry^5^, Blakemore, *et al*.^6^, Blakemore, *et al*.^7^ and Frith^8^. However, it seems that the comparisons of efferent and afferent signals alone cannot completely explain certain aspects of agency^3^,^9^,^10^,^11^. Some amputees express a feeling of agency towards a moving hand that is reflected in a mirror, seen in a location where their amputated arm would be reflected^12^. As another example of false attribution of agency Wegner, *et al*.^13^ described an experiment where participants looked at themselves in a mirror, but where the arms of the experimenter were arranged so that it could seem as if they were the arms of the participants. Participants tended to attribute a degree of authorship of the arm movements of the experimenter to themselves when there had been a prior instruction to carry out the movement, showing that priming can affect the sense of agency, even in the absence of self-executed movements.

In a recent between-groups study by Banakou and Slater^14^ immersive virtual reality (IVR) was used to embody participants in a life-sized virtual body using a head-tracked, stereo head-mounted display (HMD). The body was seen from first person perspective (1PP) as spatially coincident with and substituting their own real body. Real-time motion capture was employed to make the virtual body move synchronously with and corresponding to real body movements (visuomotor synchrony), or for another group of participants the body moved independently of real movements (visuomotor asynchrony). They saw their virtual body both by directly looking down towards their real body, and also reflected in a mirror. At some point the virtual body spontaneously spoke some words in a voice that had a higher fundamental frequency than the voices of the participants. Those in the visuomotor synchronous condition overall reported a greater sense of agency over the speaking than those in the asynchronous condition. Moreover, when after the experiment participants were later asked to speak, the fundamental frequency of their voices tended to move towards the (higher) pitch of the virtual body’s voice amongst those in the synchronous condition only. Since visuomotor synchrony was associated with much greater levels of subjective body ownership over the virtual body, the illusory perception that the virtual body was their own, it was concluded that such illusory agency could result from body ownership. In other words participants have the illusion that since the virtual body is perceptually experienced as their own, and since that body is speaking, then it must have been the participants who were speaking (since under conditions of normal health we never have the illusion that we are the authors of someone else’s utterances).

Finally, in another recent study using IVR by Tieri, *et al*.^15^ participants were embodied in a virtual body and passively observed the movements of a virtual limb that was either partially occluded or disconnected from the hand. Body ownership and vicarious agency were both decreased by visual discontinuity suggesting that visual continuity of the body limbs with the body is important for the false (vicarious) attribution of agency in the absence of real movements.

In the present study we explored how perspective view can influence illusory agency in a different domain – showing that participants can have the illusion of walking, when in fact they are seated and immobile (apart from head movements) in a fixed chair. Locomotion, such as walking, and movements of single body parts (i.e. upper limbs or the act of speaking) are different with respect to the sensorimotor mechanisms that are involved. While walking we generate bilateral cyclic movements, and although we usually initiate a gait cycle in order to reach a goal, the actual leg movements are considered highly automatic and rarely immediately goal-directed^16^,^17^, as opposed to leg movements like kicking or jumping. An additional difference between locomotion and body-part movement, is that locomotion also triggers internal sensory systems, such as vestibular sensations, as well as a perception of visual changes (optic flow) in the surrounding extrapersonal space - see the review by Patla^18^. Recent studies have suggested that when the viewpoint of a participant in IVR oscillates to generate the optic flow that would be produced by a real walk, the sensation of walking might improve^19^,^20^.

Visual feedback of a walking body that represents the participant can also produce an illusory agency effect, without the feedback from the optic flow from the surrounding environment. For example, Moseley^21^ and Soler, *et al*.^22^ tested the analgesic effect of virtual walking (by using a screen that shows a lower body walking connected with a mirror showing the real upper body moving) on paraplegic patients with neuropathic pain due to spinal cord injury. Although the overall ratings of the illusion of walking were not very high, the fact that there was a decrease of neuropathic pain after exposure to virtual walking suggests that the visual feedback of the walking motions may have been effective in correcting a mismatch between motor output and sensory feedback.

In order to test the effects of perspective and body ownership on the feeling of agency over walking movements, we immersed participants in a virtual environment, where a virtual body, seen either as spatially coincident with the real body and from a first person perspective (1PP) or separate from the viewpoint of the real body from third person perspective (3PP), was walking forward across a field (Fig. 1). Moreover, in order to test the importance of the optic flow, a second factor was whether a sway animation was applied or not to their viewpoint. This sway was based on a pre-recorded animation of real walking. In other words there was a sway applied to the head as one factor (Head Sway) that had two levels Sway or NoSway. The experiment is illustrated in Supplementary Video S1 which shows the Sway condition, and Supplementary Video S2 which shows the NoSway.

We expected a stronger sensation of body ownership and of illusory agency towards the walking movements in the 1PP. Although we expected a greater feeling of walking due to the sway movement^19^,^20^, we did not have a prediction one way or the other about its effect on agency. We evaluated body ownership and agency over the illusory walking with a questionnaire and with physiological responses to walking up a hill during the walking experience in the virtual environment. Previously physiological responses have been correlated with subjective body ownership in response to a threat^23^,^24^,^25^,^26^,^27^,^28^. However, in our case, we were interested in physiological response to preparation for a physical effort that is required to climb up a hill, since previous studies in motor imagery have shown that physiological reactions can increase during mental effort due imaginary walking or running^29^. Hence, for this study, we hypothesized that the reaction would be stronger when participants feel illusory agency and ownership towards the walking body than when this does not occur.

## Results

### Questionnaire scores

The questionnaire is shown in Table 1 (see also Methods) with results in Fig. 2, grouped by a categorisation related to ownership, agency, the experience of movement, and state. Examining Fig. 2A it is clear that 1PP resulted in greater scores than 3PP for the sensation of location at the virtual body and the illusion of body ownership, whereas with respect to the feeling that the body was that of someone else the scores are reversed (higher for 3PP). It is also clear that if the Sway Animation factor had any effect at all it was in the case of 3PP only (for *Located* and *OtherBody*). We use mixed effects logistic regression (Stata 14 function ‘meologit’) with random effects over the individuals, and with robust standard errors (to take account of possible violations of the model assumptions). For each variable, we first fitted the full model (Perspective and Sway as main effects with interaction). If the interaction effect was not significant at 5% the model was fitted with main effects only, and any main effects that were not significant were removed and the model was refitted if anything significant remained. The results are shown in Table 2, and confirm what can be seen in Fig. 2. For the questions related to the illusion of ownership (Located, Ownership) 1PP perspective results in higher scores than 3PP perspective, whereas OtherBody has lower scores for 1PP.

The 1PP condition results in higher reported Agency which is reduced in the case of Sway (Fig. 2B). Walking is slightly more complex in interpretation because the interaction term is significant. In 1PP the illusion of walking is high irrespective of Sway, but in 3PP it is lower for NoSway. This also distinguishes Walking from Agency – the first of course concerned with the illusion of walking, but the second with authorship over the walking. Note that the overall goodness of fit of each model is very good, as shown by the Wald χ^2^ values in Table 2.

### Physiological Responses

Participants experienced the walking for 4 minutes. For the first three minutes the walking was over level ground, but then continued up a hill for 44 s. We predicted that agency over the walking would result in heightened physiological responses during the hill climbing period compared to a baseline period that started 90 s before the hill climbing and lasted for as long (44 s). We recorded skin conductance, electrocardiogram (ECG) and respiration (see Methods). Physiological data from one participant (belonging to the Sway condition) were not available due to a failure in recording. A number of individual time series are shown in Supplementary Figs S1–S16.

To compare the responses across the conditions, we used as a response variable the differences between the mean skin conductance amplitudes in the hill-climbing period and the baseline (*dSC* = *mean (Hill Climbing*) *− mean (Baseline*)) (see Methods - Response Variables).

A mixed effects ANOVA for *dSC* on Perspective and Sway and their interaction found no effect for the interaction or Sway terms (P = 0.87 and 0.96, respectively) but for Perspective P = 0.025. The result for Sway does not change when the interaction is removed, and Perspective has P = 0.005 when Sway is also removed. The coefficient for Perspective (3PP = 0, 1PP = 1) is 0.28 ± 0.10 (SE), with 95% confidence interval 0.08 to 0.48. This reflects what can be seen in Table 3: that 1PP resulted in greater change in SC than 3PP, but that there are no other effects.

For the heart rate (HR) data the response variable is the change from the mean instantaneous HR during the baseline period and the mean instantaneous HR during the hill climbing (*dHR*). Table 4 shows a likely effect of perspective, but no interaction effect or main effect of Sway. The mixed effects regression confirms that neither interaction (P = 0.43) nor Sway (after removal of interaction P = 0.73) approach significance. However, Perspective by itself has coefficient 1.6 ± 0.83, P = 0.06, with 95% confidence interval −0.08 to 3.17.

Similarly for the respiration we use the change from the baseline to the hill climbing period with respect to the number of breaths per minute (*dResp*) with results shown in Table 5. There are no significant effects.

Figure 3 shows the relationship between the subjective Ownership and the physiological responses. For skin conductance dSC (Fig. 3A) there is no relationship. In the case of dHR (Fig. 3B) it appears that greater Ownership is associated with greater change in HR but only in the 1PP condition. In fact in the mixed effects regression the interaction term has P = 0.06, providing some evidence in support of what the graph suggests. It should be noted that there are several potential outliers in the 3PP case which influence the overall fit. Considering only the 1PP case then the Spearman correlation is ρ = 0.49, P = 0.009 (n = 27). There is no significant relationship for respiration (dResp).

It might be thought that since each participant had the experience twice (1PP and 3PP, counterbalanced) that the second time there would be foreknowledge about the hill climbing that could have influenced the physiological responses. This is unlikely to be the case because of the counterbalanced design. However, including order in the fitted models discussed above shows no impact at all of this factor.

## Discussion

Previous studies have suggested that the subjective experience of agency is based on the match between the predicted or the intended outcome and the perceived outcome of an action^4^,^5^,^6^,^7^,^8^,^9^,^30^. Typically agency, therefore, is assessed with respect to actual action, although, there is some evidence that illusory agency can occur in healthy participants, since actions can be attributed to the self without being executed, but just observed^14^,^29^,^31^,^32^. Moreover, recent evidence has suggested that 1PP with visuomotor synchrony between real and virtual body movements plays an important role in facilitating illusory agency^14^. Our results extend these findings, showing that even while participants are seated in a chair and not walking, seeing the virtual body from a first person perspective when it is walking can result in high levels of body ownership and self-attribution of the walking action. This is supported by both subjective and physiological responses.

Vection (optic flow indicating forward movement) was present in all conditions^33^, and correspondingly it was subjectively reported as being approximately the same on the average in all conditions (Fig. 2). This serves to validate the setup. Moreover, manipulations of the head sway to simulate walking negatively influenced the sense of agency, though at the same time enhanced the feeling of walking, in the 3PP condition. We examine these results considering the current theories of agency and focus on the apparent influence of seeing the animated virtual body from 1PP.

Although the sense of agency has been suggested to principally arise due to internal motor signals while generating a movement (internal forward models)^30^,^34^, recent studies have investigated the role of intention, suggesting that the sense of agency might also be affected by external cues that are responsible for the selection of an action - see the review by Chambon, *et al*.^9^. In other words, intentions prior to action might play a significant role in action attribution, when reafferent (visual, motor, or proprioceptive) signals become available and match with intentions in a retrospective way^35^, or even prior to action execution in a prospective way^36^,^37^. Moreover, the close match between prior intentions and subsequent action might create feelings of agency even in the absence of efferent signals (i.e. after involuntary movements)^38^, or even in the complete absence of movements^13^. Moore, *et al*.^38^ further found that in the case of involuntary movements, external cues from intention priming sufficiently close in time to the action (1 s before the movement) play a greater role in affecting judgements of agency, than in voluntary movements. Although explicit priming was not part of our study, it is possible that an intention to walk might have been created, a few moments after the virtual body started moving, and it follows that since walking is a highly trained motor behaviour, there could be a preparation of the motor commands that would normally be generated in order to walk towards a destination. This intention to walk could have prospectively enhanced the feeling of agency towards the seen walking movements, in a similar way as reported in the study of Wegner, *et al*.^13^. Moreover, since there were no efferent signals, intention might have been an even more effective cue for agency^38^.

This is similar to the argument by Banakou and Slater^14^ accounting for the finding that subsequent participant speech had its frequency shifted towards that of the virtual body only under the 1PP condition with visuomotor synchrony. It was argued that the strong body ownership associated with that condition led to motor planning for subsequent action of the same type (talking) and the same properties (walking). However, a subsequent study using the same equipment and 1PP setup, but where ownership over the virtual body was induced by visuotactile stimulation rather than visuomotor, also resulted in a high level of subjective agency over the talking, but did not result in a shift of fundamental frequency towards that of the voice of the virtual body. Hence it appears that 1PP induced body ownership alone cannot account for these results but rather they could be accounted for as a generalisation of agency from the act of moving (true agency over the movements of the virtual body) to the act of talking (illusory agency over the virtual body talking), but with 1PP as a necessary requirement.

In the case of the current experiment we found agency over the walking in the 1PP condition, but not in the 3PP, compatible with the results of Banakou and Slater^14^. There is some evidence of physiological changes in skin conductance and heart rate due to going up the hill, and some evidence that the HR change correlates with subjective body ownership, but further study is needed on this aspect. We may have found more convincing physiological results had participants initially had a period of visuomotor synchrony - i.e., where they moved and saw the virtual body move synchronously and correspondingly.

However, this discussion also highlights an important difference from the setup of Banakou and Slater^14^ since from the outset participants saw their virtual body doing something that they were definitely not doing, whereas in the earlier study the talking occurred only after several minutes into the experiment. Agency only on the basis of 1PP is unlikely. This might be explained by the work of Patla and colleagues who explored the importance of vision of the body while walking^18^,^39^. According to their results, viewing the limb position and movement plays an important role in planning and regulating the swing limb trajectory. Given this notion, it is possible that when the legs of the collocated virtual body (1PP) were observed while walking forward, an action representation for the planning of the next movement might have been initiated. Actually this is not surprising at all, since in our whole life when we look down and see our legs walking, we are walking. Hence, it is possible that a combination of the seeing the walking legs plus possible intention created by the walking experience contributed to the illusory agency. Further studies will need to carefully investigate this possibility and the relative importance of the two.

The role of visual information over proprioception on the feeling of false agency attribution has been also discussed in the past. Viewing one hand moving in a mirror, in a setup that gave the illusion that the opposite hand was moving, generated cortical motor activity related to the non-moving hand^40^ and vicarious agency seems to occur in view of an anatomically plausible structure and position of a moving arm^13^,^15^. Nevertheless, we should not disregard the influence of proprioception on body and movement perception. Goodwin, *et al*.^41^ and others^42^,^43^,^44^ have shown that directly changing proprioceptive signals through the application of vibratory stimuli to the arm’s muscles can produce illusions of movement. See the review by Proske and Gandevia^45^ on the relation of proprioception and movement perception.

Our results demonstrate the perceived sense of control over the walking movements, using explicit judgement of agency, but also there is some evidence for physiological measures - see Synofzik, *et al*.^10^ for a discussion of explicit judgements and unconscious internal feeling of agency. Although physiological responses have been correlated with subjective body ownership in response to a threat^23^,^24^,^25^,^26^,^46^,^47^, especially when the body is seen from 1PP^25^,^26^,^47^, the stimuli in our case was designed to reflect the effort of hill climbing. Results indicate a possible greater reaction to climbing the hill when the body is perceived from a 1PP. This could be related to results from studies in motor imagery, where participants imagine themselves performing a movement (from 1PP), without moving the body parts involved. Decety, *et al*.^48^ found that physiological reactions during mental effort increased, suggesting that the central programming structures might anticipate the need for energetic mobilization, when planning a movement. We argue that this could have been the case for observing (instead of imagining) from a 1PP and potentially planning the walking movements.

It is possible that our results are tied to the specific task. Walking is a special type of action that involves the entire body and it is characterized by rhythmic, alternating movement of the limbs, while walking patterns appear to be innate^16^ (for a review see Rosenbaum^49^, Chapter 5). Moreover, rhythmic activity of the leg muscles has been shown to occur in cats with disconnected spinal cord from the brain or when sensory feedback was prevented, implying that the brain is not vital for generating basic walking patterns, while there are similar but indirect observations for humans (for a review, see Dietz^50^). We predict, following Wegner, *et al*.^13^, that if participants were presented with a random or less automated action instead of walking, it would not have been possible to attribute the observed movements, at least without explicitly priming the intention before the seen action. This is an issue for further study.

A further hint about the role of embodiment on walking perception comes from a recent study by Leonardis, *et al*.^51^. Here vestibular, proprioceptive and visual stimuli were provided to participants who were not executing any movements, in order to simulate walking movements through a walking virtual body. Compared to visual stimulation only (without vestibular and proprioceptive stimulation) participants reported a greater feeling of walking and exhibited increased mean skin conductance responses and respiration rate. Although the authors relate these results to embodiment (using a combination of questions related to factors such as body ownership and sense of agency), there was no direct evidence of the establishment of the body ownership illusion (i.e. the questionnaire scores were low), and they did not discuss their results in relation to agency. However, their results provide another indication that illusory walking can occur through embodiment in VR, without the need for actual action execution.

Results from the reported feeling of walking are inline with the results from the work of Lécuyer, *et al*.^19^ and Terziman, *et al*.^20^ Our participants reported a greater feeling of walking in the swaying condition (only when in 3PP), as in those previous studies, where there was no body representation. However, it seems that the effect of the swaying camera is overridden when there is embodiment.

With respect to the influence of embodiment on the experience of agency this was considered by Caspar, *et al*.^52^. They suggested that visual incongruence of finger movements of an embodied robotic hand (the robot moves a different finger than the one that the participant moves) does not entirely abolish the sense of being the agent of an action, although it is known that a passive movements condition does abolish agency. Hence, they concluded that information from incongruent versus congruent embodiment can partially contribute to the experience of agency.

It is also important to explain at this point, the interesting result that body ownership was induced at all, using our setup. It is known that congruent combinations of sensory input from vision, touch, motor control and proprioception are some of the key mechanisms to body perception (for a review see^53^,^54^,^55^). Most studies specifically suggest that incongruent visuomotor stimulation can significantly diminish^56^,^57^,^58^,^59^, or break the illusion of ownership^60^. Thus, one would expect that since the participants did not produce any movements except head movements, it would not be possible to feel ownership towards the walking virtual body. However, recent evidence suggests that when the ownership illusion is strong, incongruent cues are not experienced as incorrect^47^, and for some cases this seems to be true (up to a point) even in the complete absence of motor execution^15^. 1PP seems to clearly dominate as an explanatory factor for subjective and physiological measures of ownership^46^, while congruent head movements are also essential when other cues are not so dominant^26^,^47^. It seems possible that these two cues alone could override incongruent visuomotor stimulation of body parts when there is visual continuity of the body^15^ and our results suggest that this could also be possible in the case of walking.

## Methods

### Experimental Design

Twenty eight participants (20 female; mean age 23 ± 3.5 years) were recruited by advertisement around the University campus. The experiment was approved by the Comissio Bioética of the University of Barcelona and carried out in accordance with the approved guidelines. All participants gave written informed consent and were paid 5 euros for their participation.

The experiment had a 2 × 2 mixed effects design with one between factor (Head Sways) that had two levels (NoSway and Sway) and one within factor (Perspective Position) with two levels - third person perspective (3PP) and first person perspective (1PP). In other words, participants were in two groups where one group experienced the walking experiment with Sway and the other group with NoSway. The order of the within factor was counterbalanced across the participants within each group. In the 1PP condition participants saw a life-sized virtual body spatially coincident with their own (obscured) body, and seen from a first person perspective as if from the eyes of that body. In the 3PP participants saw the same virtual body but outside of and slightly in front and to the left of themselves.

**Figure 1:**
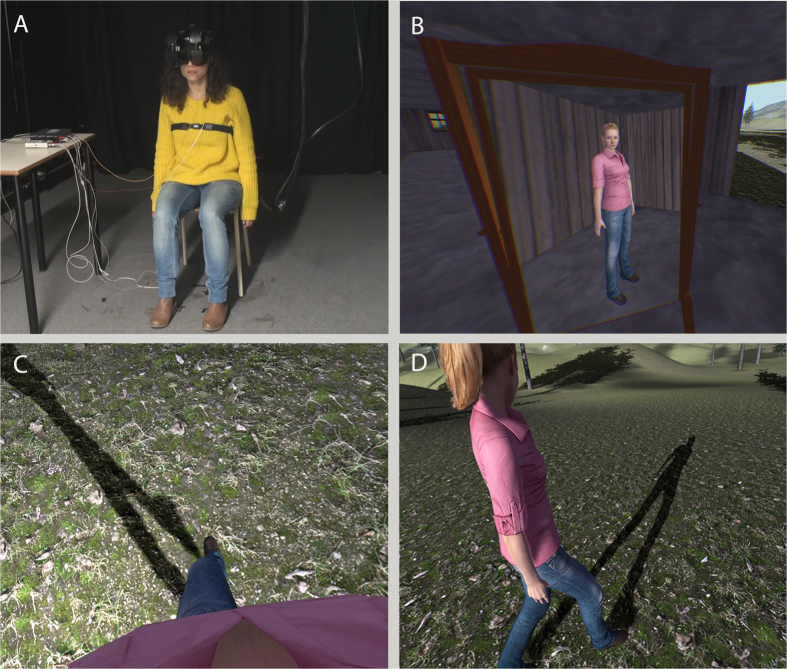
Illusory Walking setup. (**A**) Participants were seated still on a stool, except for head movements. (**B**) Initially participants saw the standing virtual body reflected in a mirror (1PP condition). (**C**) Participants saw the walking virtual body from 1PP, or (**D**) from 3PP. The walking body always cast a shadow.

**Figure 2:**
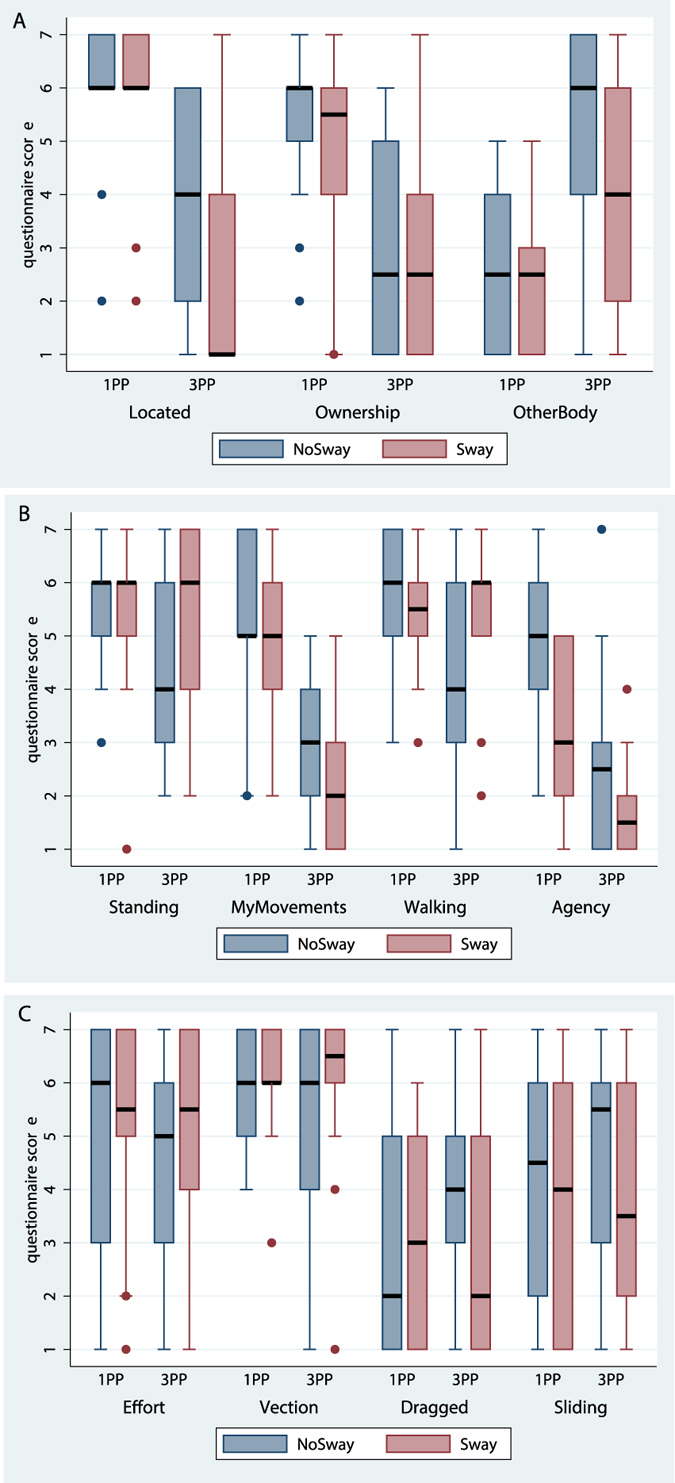
Box plots of the questionnaire results. (**A**) The body ownership questions. (**B**) Related to agency. (**C**) Related to experience of movement. (**D**) Related to state - walking and effort.

**Figure 3:**
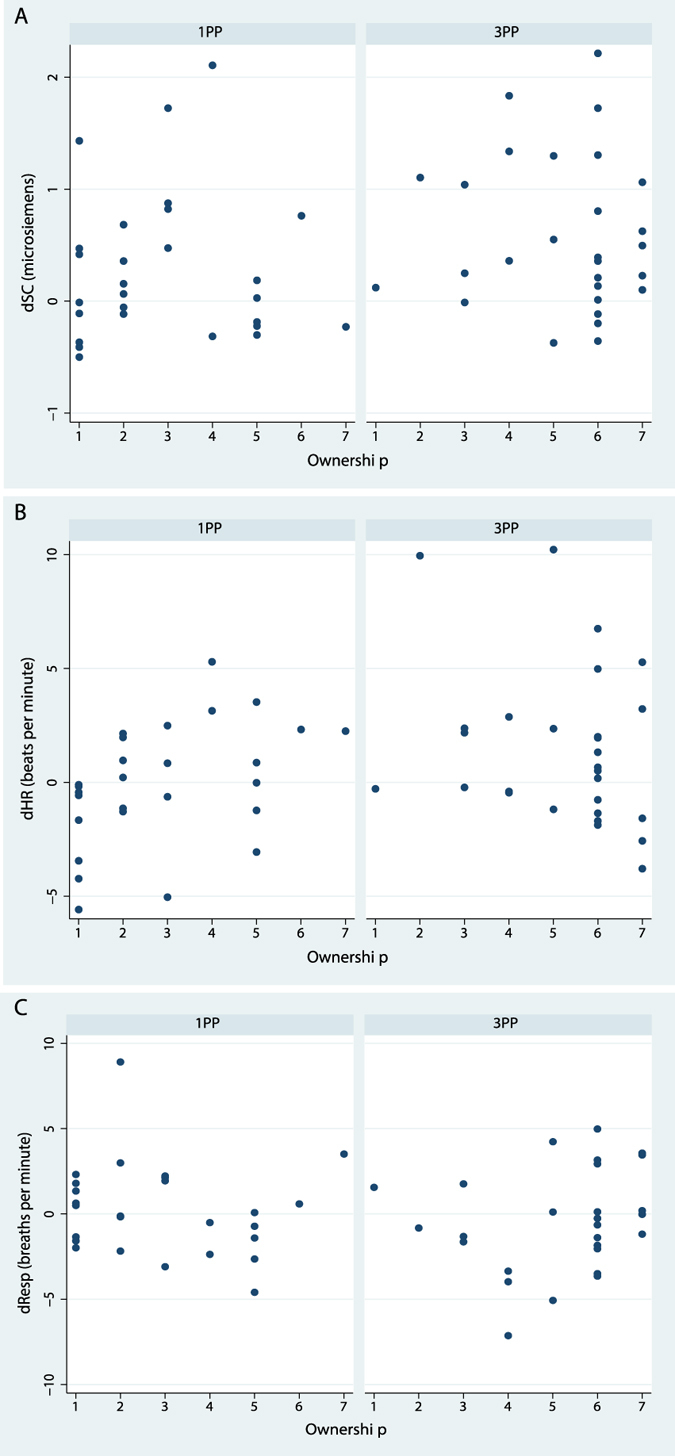
Scatter diagrams of change in physiological responses from baseline to hill climbing by Ownership. (**A**) For skin conductance (dSC), (**B**) for heart rate (dHR) and (**C**) for respiration (dResp).

Seated participants donned a head-tracked HMD (Fig. 1A), so that their view was always updated as a function of their head orientation. In the Sway condition their view was further modulated by a walking animation as applied to the head (in other words swaying slightly from side to side and up and down). After participants were immersed in the virtual world, their point of view moved forward with a spatially coincident (1PP) or a non-spatially coincident (3PP) walking virtual body (Fig. 1C,D). By spatially coincident we mean that the virtual body visually substituted the person’s real body, albeit the virtual body was standing while the real body was seated. Depending on the Sway Animation condition, their point of view would additionally follow the walking oscillations, following the pre-recorded walking animation (Sway), or just following the main axis of the forward movement without any oscillations (NoSway). It could be expected that the foreign walking and swaying movements would produce additional motion sickness. A few participants reported mild nausea (but not severe), which is not unusual for many VR applications that involved movement through a space.

### Materials

Participants were immersed in the virtual reality scenario by fitting them with a stereo NVIS nVisor SX111 HMD. This has dual SXGA displays with 76° Hx64° V field of view per eye, with 50° (66%) of overlap, totalling a wide field of view of 102° horizontal and 64° vertical, with a resolution of 1280 × 1024 per eye displayed at 60 Hz. Head tracking was performed by a 6-degrees of freedom Intersense IS-900 device. Audio feedback was provided through headphones to further isolate participants from sounds from the laboratory.

The virtual environment was implemented using the Unity3D platform. The virtual model of the room was designed in 3D Studio Max 2010, and we used animation-enabled models of male and female virtual bodies purchased from Rocketbox Studios. The walking animation for the virtual body was recorded using an Optitrack Motion Capture system, and manually refined using Autodesk Motion Builder 2012 software.

Physiological (electrocardiogram (ECG), skin conductance and respiration) signals were recorded at a sampling rate of 256 Hz, using the g.tec bio-signal acquisition device g.USBamp, while recordings and storage of the data were handled by a Simulink model in Matlab. All statistical analysis was carried out with Stata 14.

### Procedures

#### Preparation

The participant was seated on a stool in the VR lab (Fig. 1A). Participants were told that they would be immersed in a virtual environment and that they would see a virtual body, without specifying whether the body would be seen from a 1PP or a 3PP. They were also told that this body would start moving forward, without specifying that the body would perform walking movements. After the experimenter verbally gave the instructions and attached the sensors for recording the physiological signals, the participants donned the HMD and headphones and they were immersed in the virtual environment, providing them 1PP or 3PP view of a gender-matched virtual body that was in a standing position. The HMD was calibrated for each participant using the method described in ref. 61.

The displayed scene was initially the inside of a wooden cabin from which a mountain landscape could be seen through the windows and the open door (Supplementary Video S1). There was also an ambient sound of birds appropriate for the environment, which served to isolate participants from any lab sounds. Participants were first instructed to describe the virtual world, in order to familiarize themselves with the environment and with the virtual body, which could also be seen as a reflection in a virtual mirror (Fig. 1B). They were also instructed not to move any part of their body except for their head, throughout the experiment.

#### Walking Phase

After the familiarization phase, the virtual body started walking forward, towards the mountain landscape, outside the wooden cabin. The virtual body walked for 4 minutes at a constant pace, using a pre-recorded animation applied to the entire body, excluding the arms. Each complete walk cycle would last 1.267 s, which resulting in a constant velocity of 1 m/s. The viewpoint of the participants followed the virtual body at the same speed, so as to be always seeing the body from 1PP (from a position corresponding to the eyes of the virtual body) (Fig. 1C) or from a constant distance of 20 cm to front and 100 cm to the left (3PP) of the virtual body (Fig. 1D). We chose this as a distinguishable distance between participant’s viewpoint and the virtual body location. In the case of the NoSway condition, the viewpoint corresponded to that based on the head tracker for orientation and followed the virtual body’s forward movements for position. However, in the case of Sway a further animation was applied to the viewpoint, simulating a head sway corresponding to a walking motion. This was accomplished by allowing the viewpoint to follow the oscillations of the virtual body’s head produced by the pre-recorded walking animation.

In order to control for the amount of time that each participant was looking towards the body and specifically towards the walking legs, virtual balls appeared at random points 12 times along the walking path. Each ball appeared 6 meters in front of walking body and participants were instructed to look towards the virtual legs for a few seconds every time they would see one of the balls. These were either the legs seen from the 1PP body or from the 3PP body. However, as in any experiment where the participants’ eyes are not visible to the experimenter and not eye-tracked, it is not possible know the exact time that participants spent looking at the stimuli, nor how much this varied across participants.

On the third minute of the virtual walk, the virtual body approached and started climbing a 32 m long hill with 20° of inclination, using the same walking animation. The hill climbing lasted 44 s. We assumed that this would be perceived as a demanding walking task and we expected physiological responses to this activity. The walk continued for another 21.5 m until a stop sign was reached, at which moment the experiment was concluded.

The task was repeated twice; once for the 1PP and once for the 3PP condition, with a 5 min break between where participants completed a questionnaire. After the second repetition they completed an identical questionnaire, were briefed about the purpose of the experiment and were compensated for the participation.

### Response Variables

#### Questionnaire

After each Perspective trial (1PP, 3PP), a questionnaire was given to the participant. This was designed to assess the level and quality of (a) the body ownership illusion experienced by the participants, derived from^62^, and (b) the level of the experienced feeling of walking and agency over the walking movements. Participants were asked to rate 11 statements appearing in a different random order for each participant (Table 1).

The first question (*Located*) referred to self-localization. *Ownership* is concerned with the subjective strength of the ownership illusion whereas *Otherbody* is a corresponding control question. *Standing* assessed the feeling of standing (instead of seating on a chair). *MyMovements* was concerned with the sense of ownership of the movement, whereas *Agency* was concerned with the sense of motor control^63^. *Effort* assessed the feeling of extra effort when going up the hill. The remaining questions concerned the feeling of moving: *Vection* moving in space; *Walking* the feeling of walking; *Dragged* the feeling of being dragged, and *Sliding* the feeling of sliding. *Dragged* and *Sliding* were exploratory items in order to understand participants’ perception of the walking movements (i.e. more passive or active).

#### Physiological Responses

Physiological activity during mental effort in motor imagery studies has been shown to increase beyond the level of metabolic demands of a static body, suggesting that the central programming structures might anticipate the need for energetic mobilization, when planning a movement^48^. We hypothesized that if agency would occur during the virtual walking, then participants would react physiologically to the demanding task of walking uphill. We recorded skin conductance, ECG and respiration throughout the experiment. Our purpose was also to find out whether these were influenced by the different experimental conditions. Heart rate (HR) and respiration rate (RR) were calculated as the mean instantaneous HR or RR (reciprocals of the HR or RR intervals) during two periods: the stimulation period (climbing the hill) and a baseline period. We chose the baseline period to start 90 s before reaching the hill, because we assumed that there would be no physiological reactions from changes in the environment from that moment. Since the overall time to climb the hill was 44 s, the time duration of both stimulation and baseline periods was 44 s. Similarly we calculated the maximum amplitude of skin conductance levels during the same baseline and stimulation period.

## Additional Information

**How to cite this article**: Kokkinara, E. *et al*. First Person Perspective of Seated Participants Over a Walking Virtual Body Leads to Illusory Agency Over the Walking. *Sci. Rep.* **6**, 28879; doi: 10.1038/srep28879 (2016).

## Supplementary Material

Supplementary Information

Supplementary Information

Supplementary Information

## Figures and Tables

**Table 1:** The post-experience questionnaire.

Question	Statement
*Located*	During the experiment I felt as if my body was located where I saw the virtual body to be.
*Ownership*	During the experiment I felt that the virtual body was my own body.
*Standing*	During the experiment I felt that I was standing upright.
*MyMovements*	During the experiment I felt that the leg movements of the virtual body were my movements.
*Agency*	During the experiment I felt that the leg movements of the virtual body were caused by my movements.
*OtherBody*	During the experiment I felt that the virtual body belonged to someone else.
*Effort*	I felt I had to give extra physical effort when I reached the hill.
*Vection*	I felt that I was moving through space rather than the world moving past me.
*Walking*	I felt that I was walking.
*Dragged*	I felt that I was being dragged.
*Sliding*	I felt that I was sliding.

All questions were rated on a Likert scale from 1 (totally disagree) to 7 (totally agree).

**Table 2:** Mixed effects logistic regression of the questionnaire responses.

Question	Perspective (3PP = 0, 1PP = 1)	Sway Animation (NoSway = 0, Sway = 1)	Interaction	Wald χ^2^	d.f.	Overall P
*Located*	3.6 ± 0.85 (0.000)			17.83	1	0.0000
*Ownership*	2.4 ± 0.63 (0.000)			14.37	1	0.0000
*Standing*	–					
*MyMovements*	4.1 ± 0.57 (0.000)			50.1	1	0.0000
*Agency*	4.4 ± 0.73 (0.000)	−3.2 ± 1.45 (0.028)		37.98	2	0.0000
*OtherBody*	−1.9 ± 0.59 (0.002)			9.86	1	0.0017
*Effort*	–					
*Vection*	–					
*Walking*	2.5 ± 0.59 (0.000)	1.9 ± 1.10 (0.087)	−2.7 ± 1.00 (0.006)	18.20	3	0.0004
*Dragged*	–					
*Sliding*	–					

The entries are coefficient estimate ± S.E. of the corresponding effect in the linear predictor (with the significance level in brackets). 0.000 means P < 0.0005, and 0.0000 means P < 0.00005. The – symbol indicates no significance.

**Table 3:** Means and SEs of change in skin conductance (microsiemens) from baseline to hill climbing (*dSC*).

	NoSway	Sway	Overall Mean
3PP	0.28 ± 0.20	0.29 ± 0.16	0.29 ± 0.13
1PP	0.58 ± 0.21	0.60 ± 0.16	0.59 ± 0.13
Overall Mean	0.43 ± 0.15	0.45 ± 0.12	0.44 ± 0.09

**Table 4:** Means and SEs of change in instantaneous HR (beats per minute) change from the baseline to the hill climbing.

	NoSway	Sway	Overall Mean
3PP	−0.55 ± 0.74	0.39 ± 0.69	−0.10 ± 0.51
1PP	1.62 ± 0.91	1.28 ± 0.97	1.45 ± 0.65
Overall Mean	0.54 ± 0.61	0.85 ± 0.60	0.69 ± 0.42

**Table 5:** Means and SEs of change in respiration rate (*dResp*) from the baseline to the hill climbing.

	NoSway	Sway	Grand Mean
3PP	−0.19 ± 0.50	0.67 ± 0.92	−0.22 ± 0.51
1PP	−0.13 ± 0.81	−0.70 ± 0.78	−0.42 ± 0.77
Grand Mean	−0.16 ± 0.47	−0.04 ± 0.60	−0.10 ± 0.38
