# Supplementary material for: First Person Perspective of Seated Participants Over a Walking Virtual Body Leads to Illusory Agency Over the Walking
Source: Sci Rep. 2016 Jul 1;6:28879. doi: 10.1038/srep28879 (PMC4929480; doi:10.1038/srep28879)
Supplement: Supplementary Information [file srep28879-s3.pdf]

First Person Perspective of Seated Participants Over a Walking Virtual Body Leads to Illusory Agency Over the Walking

Elena Kokkkinara[1] , Konstantina Kilteni[1], Kristopher J. Blom[1], Mel Slater*[1,2]

[1]Event Lab, Faculty of Psychology, University of Barcelona
[2]Institució Catalana de Recerca i Estudis Avançats – ICREA

* Corresponding Author: melslater@ub.edu

Supplementary Figures

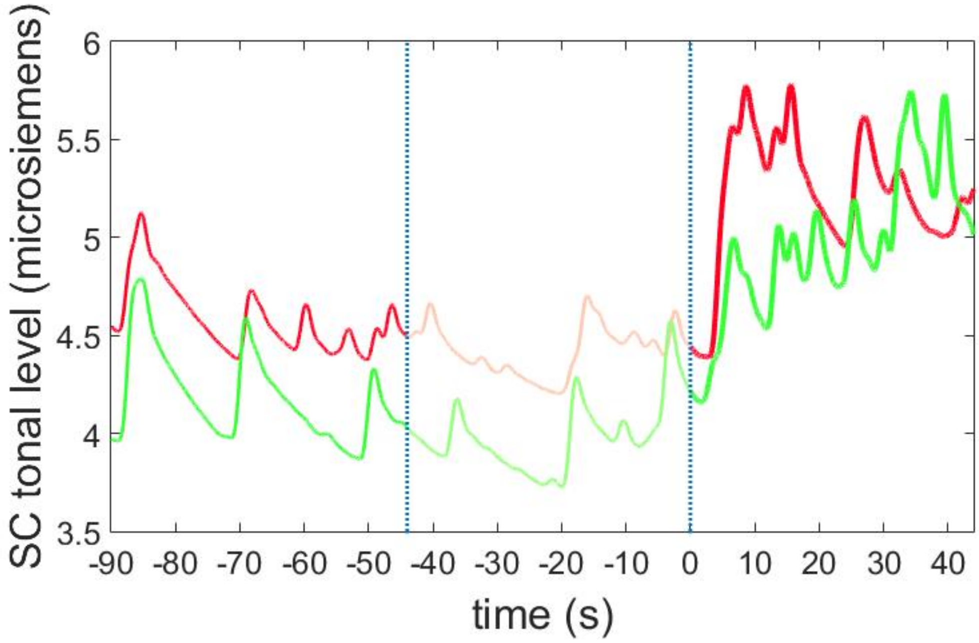

Figure S1 - Skin conductance by time for a subject in condition (NoSway, 3PP)

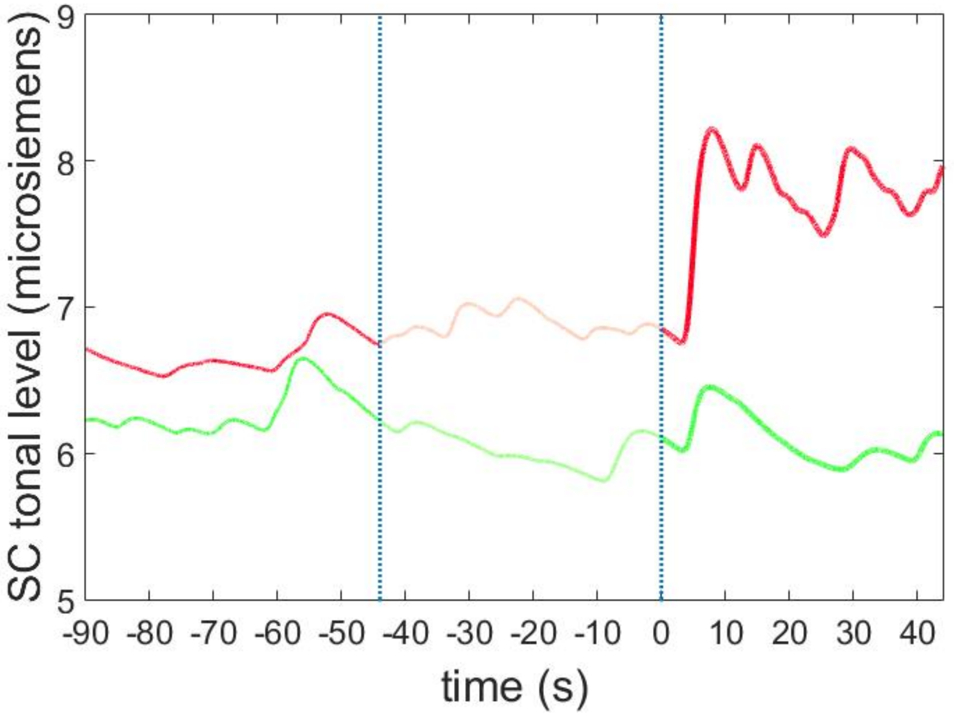

Figure S2 - Skin conductance by time for a subject in condition (NoSway, 3PP)

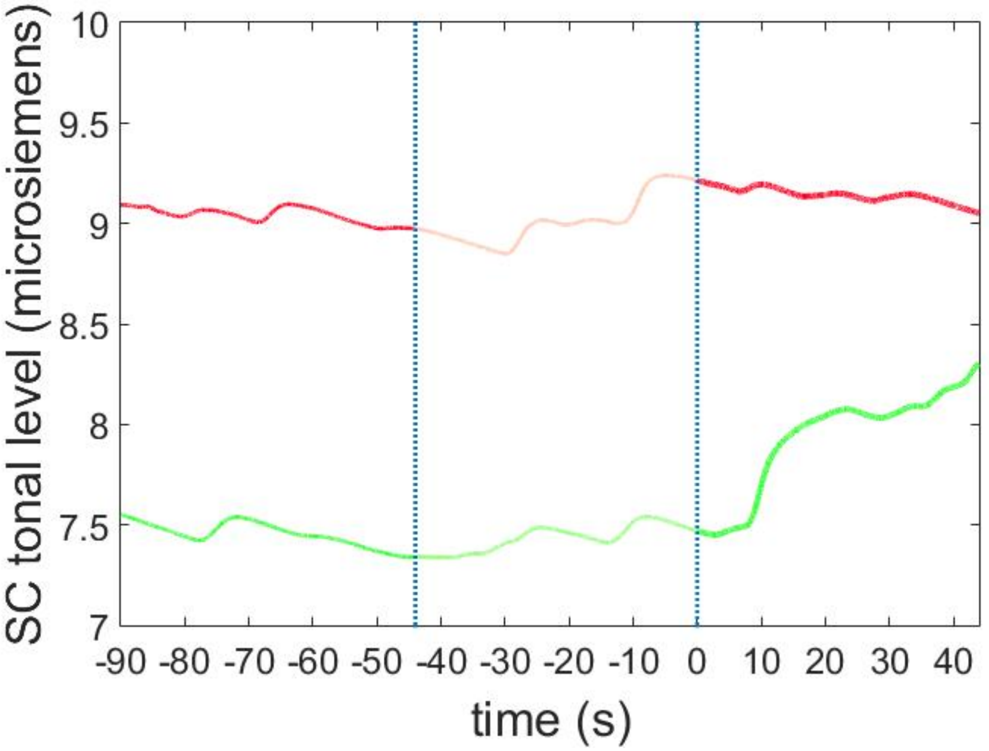

Figure S3 - Skin conductance by time for a subject in condition (Sway, 3PP)

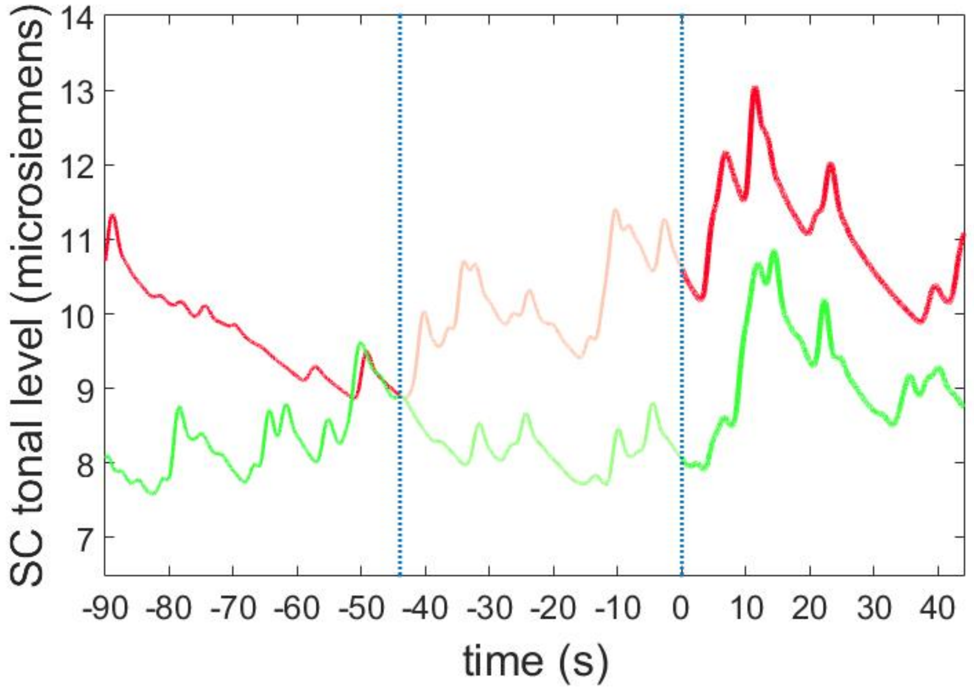

Figure S4 - Skin conductance by time for a subject in condition (Sway, 3PP)

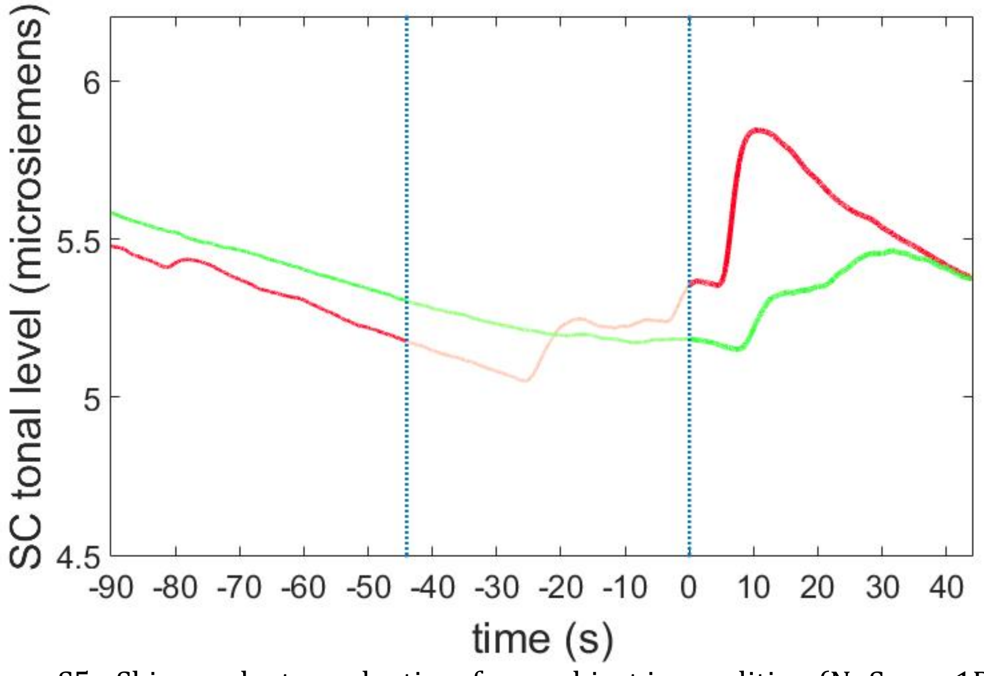

Figure S5 - Skin conductance by time for a subject in condition (NoSway, 1PP)

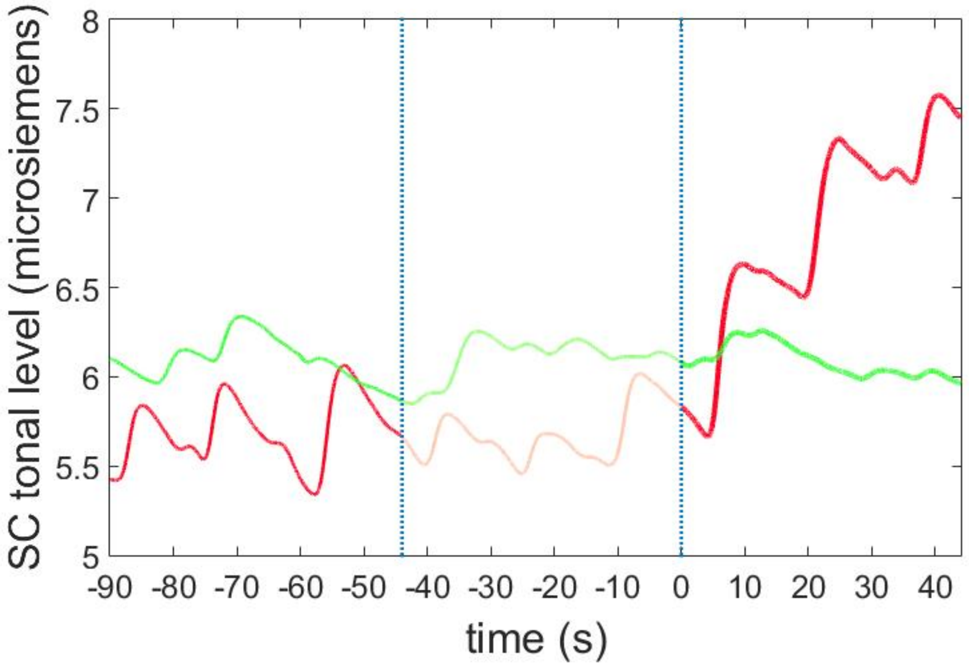

Figure S6 - Skin conductance by time for a subject in condition (NoSway, 1PP)

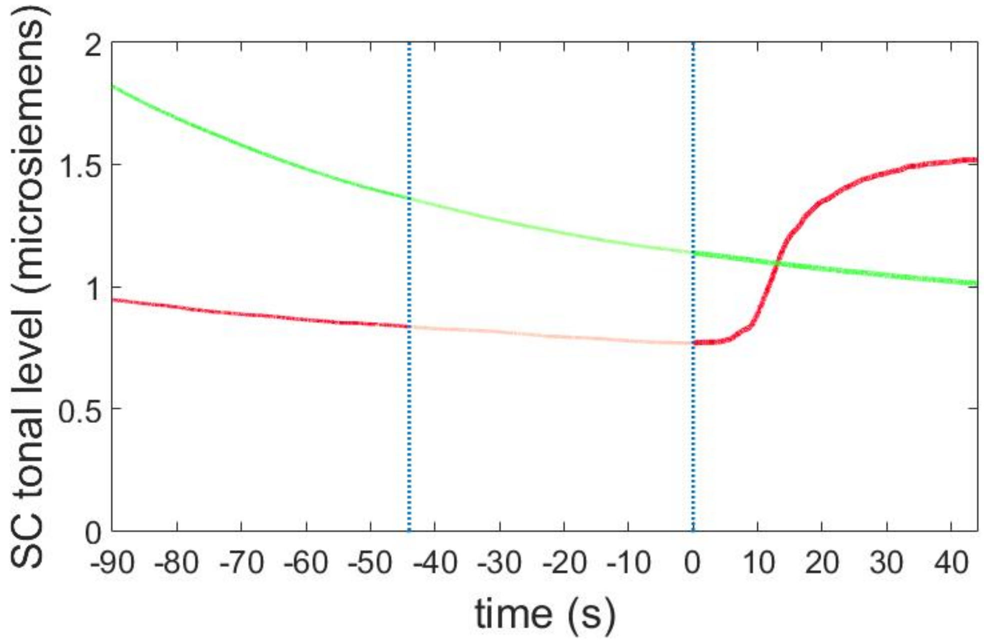

Figure S7 - Skin conductance by time for a subject in condition (Sway, 1PP)

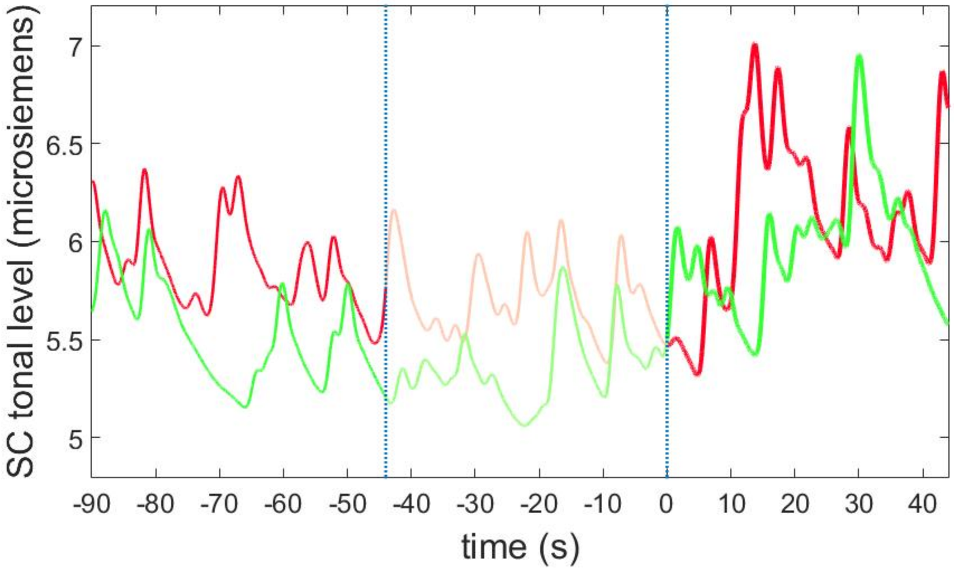

Figure S8 - Skin conductance by time for a subject in condition (Sway, 1PP)

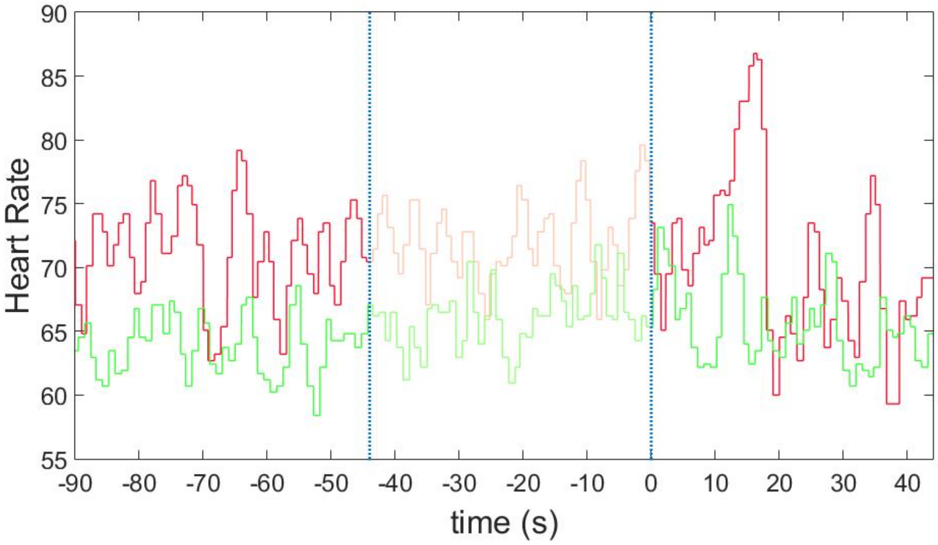

Figure S9 - Heart Rate by time for a subject in condition (NoSway, 3PP)

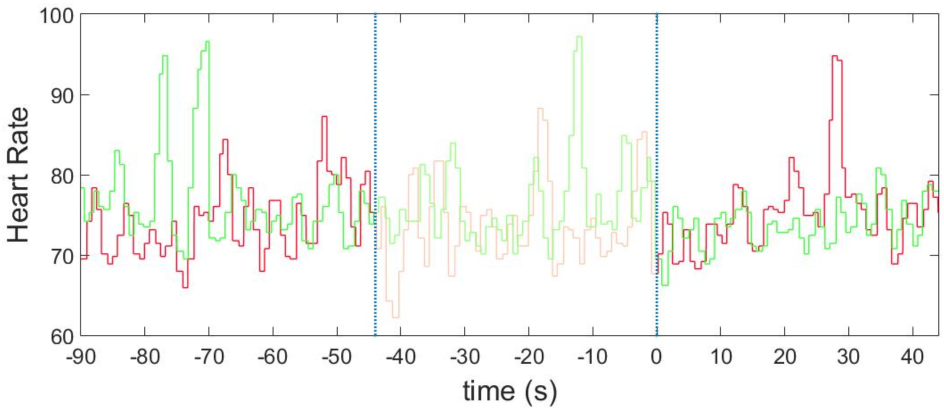

Figure S10 - Heart Rate by time for a subject in condition (NoSway, 3PP)

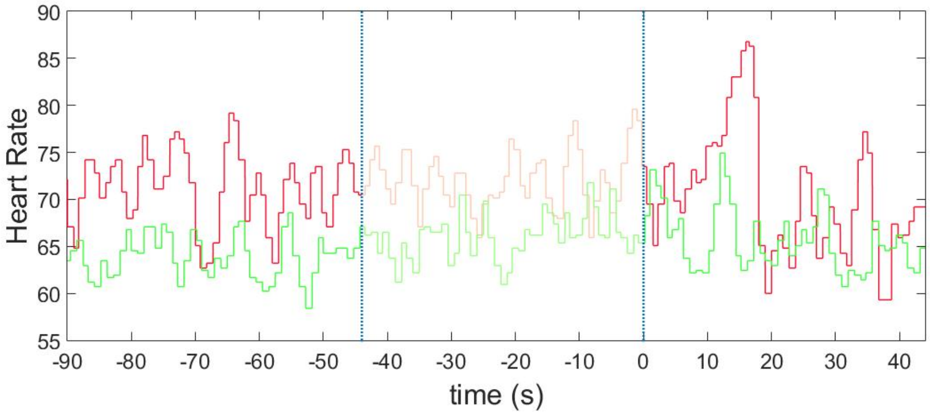

Figure S11 - Heart Rate by time for a subject in condition (Sway, 3PP)

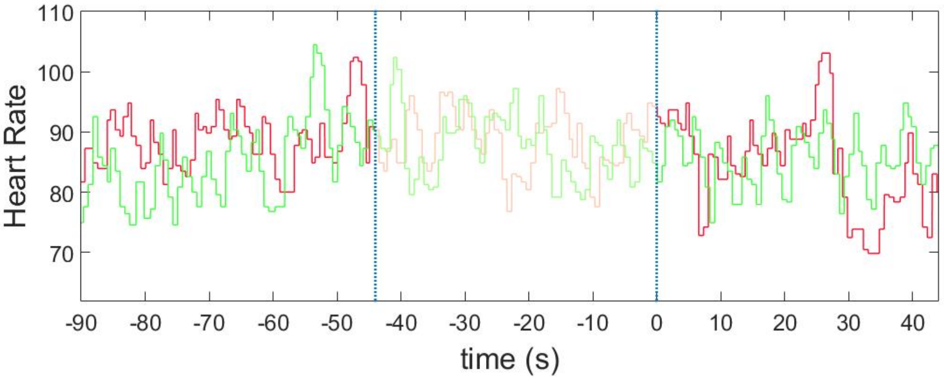

Figure S12 - Heart Rate by time for a subject in condition (Sway, 3PP)

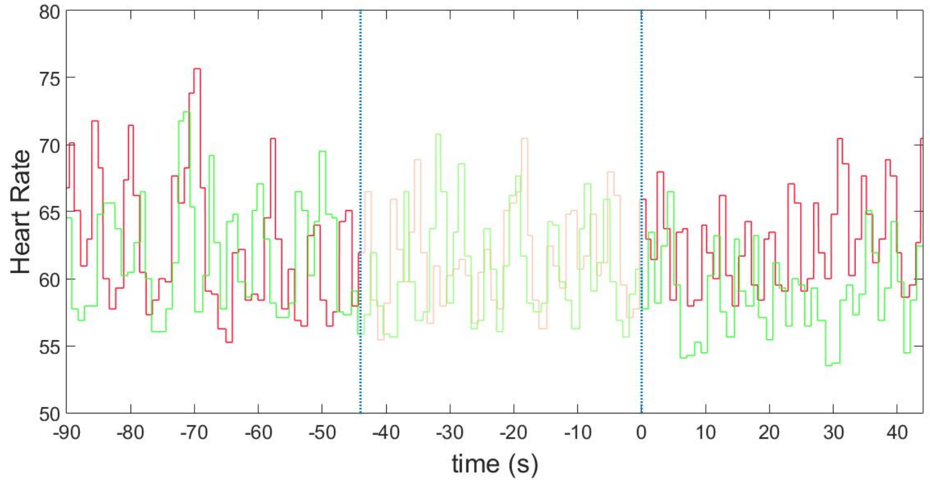

Figure S13 - Heart Rate by time for a subject in condition (NoSway, 1PP)

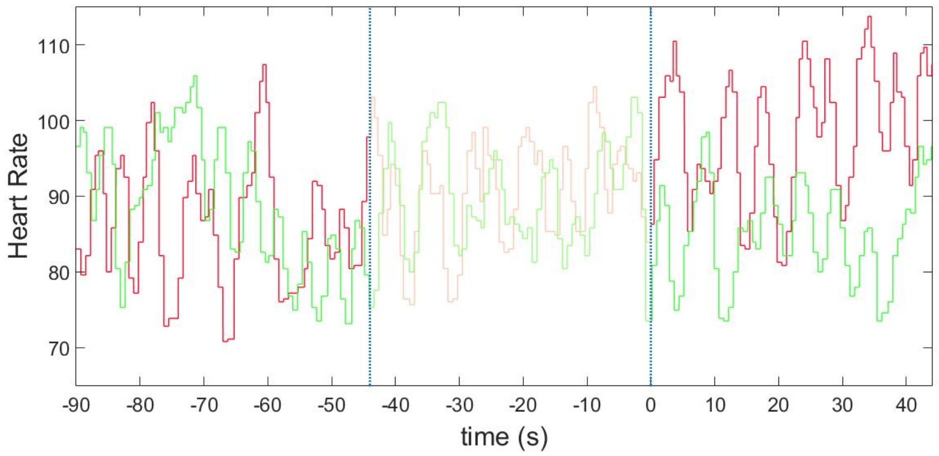

Figure S14 - Heart Rate by time for a subject in condition (NoSway, 1PP)

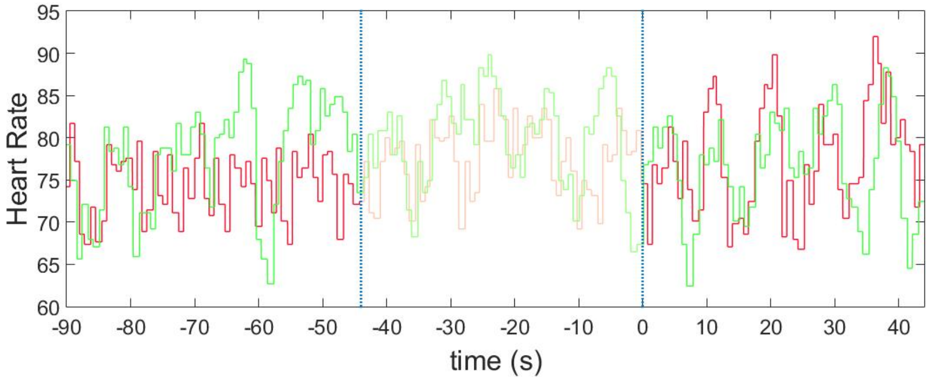

Figure S15 - Heart Rate by time for a subject in condition (Sway, 1PP)

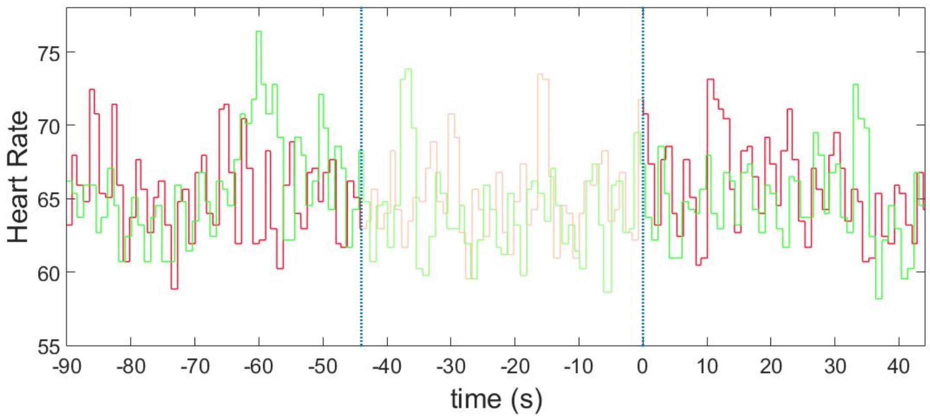

Figure S16 - Heart Rate by time for a subject in condition (Sway, 1PP)

Supplementary Video Legends

Supplementary Video S1

The complete scenario showing 1PP, 3PP and the Sway condition.

Supplementary Video S2

The scenario showing 1PP, 3PP and the NoSway condition.
